# Key insights in the AIDA community policy on sharing of clinical imaging data for research in Sweden

**DOI:** 10.1038/s41597-020-00674-0

**Published:** 2020-10-06

**Authors:** Joel Hedlund, Anders Eklund, Claes Lundström

**Affiliations:** 1 Center for Medical Image Science and Visualization (CMIV), Linköping University, Linköping, Sweden; 2 Department of Biomedical Engineering, Linköping University, Linköping, Sweden; 3 Division of Statistics and Machine Learning, Department of Computer and Information Science, Linköping University, Linköping, Sweden; 4 Department of Science and Technology, Linköping University, Linköping, Sweden

**Keywords:** Data publication and archiving, Image processing, Data acquisition

## Abstract

Development of world-class artificial intelligence (AI) for medical imaging requires access to massive amounts of training data from clinical sources, but effective data sharing is often hindered by uncertainty regarding data protection. We describe an initiative to reduce this uncertainty through a policy describing a national community consensus on sound data sharing practices.

Artificial intelligence (AI) shows great promise in many domains, and diagnostic imaging is an area where the potential is particularly articulated. The main bottleneck for achieving high-quality AI results is access to massive amounts of representative training data. Sharing of painstakingly gathered data collections is, therefore, an essential part of making substantial advances in the field^1^. Moreover, sharing is necessary for the purpose of research reproducibility^2^.

The data in diagnostic imaging, at least in their original clinical form, are sensitive personal data. The privacy of the patients must always be protected. There is now high awareness about data protection needs^3^, especially since the European general data protection regulation (GDPR) was established, but there is great uncertainty about how to interpret and implement the generic legal concepts in the practice of the domain. This uncertainty is acting as a barrier to many prospective efforts, severely hampering research and innovation in AI for medical imaging.

The Analytic Imaging Diagnostics Arena (AIDA) is a community effort engaging about 60 Swedish organizations from academia, industry and healthcare in an innovation arena for AI in medical imaging. Being a national facilitator, we have at AIDA drawn up a data sharing policy^4^ to provide succinct and understandable guidelines for activities in our community.

Here we describe the data sharing policy, to introduce this resource for researchers and medical staff in the area, with the intent that this could serve as a stepping-stone for further refinement and discussion of best practices in data sharing. We introduce the policy in two parts. The first is a discussion of how the policy was designed to be accessible to non-experts, while being legally accurate, and the second puts the policy into context regarding key sharing issues, and outlines how the AIDA policy may benefit a wider audience.

The AIDA policy complements previous efforts^1,5,6^, for example the Royal College of Radiology guidance on secondary use of patient images^7^, in relation to which the AIDA policy ventures further both into concrete practical instructions and detailed legal references. For the United States setting, protective measures for the clinical imaging domain are discussed in an ACR-AAPM-SIIM guideline^8^.

## Policy Overview

The AIDA data sharing policy comprises six sections: *Context*, *AIDA data sharing*, and four appendices on *Legal discussion*, *Ethical review applications*, *Anonymization*, and *Templates*.

The Context section is intended to be a complete introduction for readers who do not need to drill deeper into the details. It describes what the AIDA community has identified as the common practice in ethical, legal, and FAIR (findable, accessible, interoperable, and reusable) sharing of clinical imaging data for research in Sweden, with reference to official sources in law. Additionally, it provides a brief discussion on practical research ethics in light of the legal context, along with an introduction to protective measures.

The policy is intended to be a comprehensive resource that includes practical instructions for researchers interested in sharing or accessing data through AIDA. The second section, ‘AIDA data sharing’, provides such details, listing the available tools and systems, and the mechanisms employed in the sharing pipeline.

In order to increase general readability, and to be inclusive for non-specialist readers, lengthy discussions in the data sharing policy have been put into appendices. The first appendix is an extended legal discussion of seven regulations and laws relevant for sharing clinical data.

The appendix on ethical review applications is intended to support researchers in planning their data sharing activities to fulfill ethical and legal requirements. As an aid, this section contains a collection of concrete examples of approaches used in successful applications to the Swedish national ethical review authority.

The appendix on anonymization provides general guidelines for clinical imaging data, as well as concrete examples of how this has been done for some AIDA datasets.

The final appendix provides templates intended to serve as a starting point for organizations looking to set up their own agreements for legal and ethical data sharing outside of AIDA.

## Connecting Practical Instructions to the Formal Context

A common experience in the AIDA community is that when researchers and clinical staff turn to their organization’s legal advisors for guidance, the responses are generic and non-conclusive. This is natural, since legal specialists are seldom also experts on diagnostic imaging. Moreover, there is a lack of domain-specific guidelines for data sharing practices. Thus, many implementation aspects regarding data management are left to the individual researchers to define. We argue that diagnostic imaging domain experts must take the helm in interpreting the legal and ethical requirements for their data sharing. To this end, the AIDA policy is intended as a concrete and practical guide for sharing of clinical imaging data for research.

The ambition to provide user-friendly instructions may, however, negatively impact the precision with which key concepts are described. The AIDA experience is that much of the uncertainty and confusion regarding data sharing stems from an imprecise understanding of the central terms used in legal text. Therefore, in developing this policy, much effort was directed into connecting practical instructions to the formal context via precise term definitions.

Many of those definitions are emphasized in the policy’s initial description of common practice for sharing of clinical imaging data for research in Sweden:

“*In brief, the common practice is that* *caregivers disclose* *data to* *research institutions* *for specific activities described in approved* *ethical review applications*, *to be carried out under appropriate technical and organizational* *protective measures* *and supervised by a named* *competent researcher*. *The research institution is then* *data controller* *and* *copyright holder* *for the disclosed data, and is responsible for ensuring that data is processed and shared only as described in the approved ethical review application, with* *data processing agreements*, *pseudonymization*, *anonymization* *and* *licensing* *as tools, and with an obligation to store relevant data for 10 years after last use for purposes of* *research validation*.”

This paragraph comprehensively but concisely outlines the main components and key concepts of data sharing practices, with key terms hyperlinking to more detailed descriptions in the legal discussion appendix. The common practice description also reduces ambiguity by clearly defining the scope of sharing scenarios.

The term ‘protective measures’ can similarly be open to interpretation, so these have also been described with precise terminology in the context section. We have grouped these into three categories: informational, organizational, and technical measures.

The practicalities of data sharing also require a clear understanding of the roles involved, and the connected responsibilities. The policy section describing AIDA data sharing includes a definition of the roles involved in sharing a dataset: author, copyright holder, and dataset contact. The AIDA policy concludes that the research institution obtaining the ethical approval is normally considered the copyright holder.

## Key Conclusions in the Policy

In this section we highlight four AIDA conclusions that may be of interest for developing similar policies.

### Obligation to share

Ethical considerations are at the core of data sharing. While integrity issues must be considered, researchers have an ethical obligation not to let unreasonable concerns hinder investigations of societal value. This viewpoint is in line with the recently proposed ethical framework for clinical imaging data sharing^9^.

### Legal basis

An approved ethical review is a cornerstone in the common practice, and this policy clarifies its relation to GDPR in the Swedish setting. In Sweden, reviews are handled by the national ethical review authority (EPM). The legal basis for processing patient data in a research context defined by GDPR is part of the EPM evaluation. The review ascertains whether the research is of sufficient public interest, that appropriate methods and safeguards are used, and that any risks to individuals are proportionate given the aims of the study. Thus, an approval establishes the legal basis for carrying out the research.

An important note is that informed consent does not have an exceptional standing in the policy with respect to legal bases for processing. While consents can be part of the legal basis for processing, it is neither a necessary nor a sufficient condition. The experience in the AIDA community is that informed consent is often erroneously assumed to be the only legal basis admissible for data sharing.

### Anonymization definition

We have made efforts in the AIDA policy to define anonymization. Not least because this is an important area for consideration in image data sharing, but in our experience is often misunderstood, with clinical professionals using the term ‘anonymization’ to describe pseudonymization.

The policy appendix on anonymization contains examples and rationale for what AIDA considers correct anonymization. The interpretation of anonymity is central, since GDPR does not apply to anonymous data. The GDPR definition of anonymity says that re-identification must be impossible considering all means *reasonably likely* to be used. The AIDA policy pins down the following interpretation of the GDPR definition:

*“Data is anonymous when the de-identification procedure results in that there is no reasonably practical way for anyone, not even a care provider employee or IT support staff of the care provider, to reconnect the data (the image and/or the meta data) to the clinical record.”*

A fundamental question in our domain is whether the image as such, the pixels, could be considered a unique key back to itself in the clinical database. The AIDA policy concludes that images extracted from clinical databases *can* be anonymous. The rationale is that an exhaustive image-matching search in the clinical system would currently not be reasonably practical (as opposed to, for instance, a direct database query using a text string). Such an attack would require both an undetected installation of enhanced computational power and of additional bandwidth to the clinical storage systems, to run such a search without noticeably impeding clinical production. Furthermore, the AIDA policy argues that if a person would have such unabridged access to the clinical system, then all patient data in the system would be compromised, and no additional integrity risk arises from having a research copy of clinical data.

The AIDA policy also concludes that anonymization in itself is to be considered lawful, when done to share the data for ethically approved research, and that anonymization is one way to follow the data minimization principle in GDPR.

### Using cloud solutions

One part of the policy specifically looks into legal aspects of data processing services in the form of cloud solutions. The Swedish public authorities have jointly concluded that it is not possible to safeguard data confidentiality when storage providers are governed by legislation that may require data sharing with its national authorities^10^. This means, for instance, that Swedish universities are not normally allowed to process data using cloud services provided by companies originating from the United States, regardless of where the physical servers are located. This can be overruled on a case-by-case basis if the possibility can be dismissed that disclosing the data to foreign powers could lead to harm to an individual, or conflict with national interests. There is, however, an ongoing debate over this interpretation.

At the EU level, the European Commission maintains a list^11^ of countries outside the EU found to offer an adequate level of data protection, along with any limitations that apply. For the United States, the Privacy Shield framework was previously seen as adequate, but the adequacy was recently invalidated by the Court of Justice of the European Union^12,13^.

The cloud service aspect is reflected as follows in the policy, referring to the GDPR requirement on sufficient guarantees for protective measures:

*“In regards to sufficient guarantees, it may be easier for a research institution in the EU to obtain such guarantees from data processors that are based in the EU (or even in the same country) and as such themselves are regulated by GDPR, keeping in mind that data processors based outside the EU may not be legally allowed to fulfill any guarantees given, depending on national legislation in the country where they are based […]”*

## Policy Development Process

The development of the policy was a grassroots effort by a national community of researchers and other stakeholders. This group, first established in 2017, identified the uncertainty around data sharing practices as an important obstacle. Discussion was initiated at an AIDA workshop early 2018 between GDPR experts from the Swedish Data Protection Authority and the Swedish Research Council, and AIDA researchers and clinicians. This was followed by an iterative elicitation of best practices through a series of input sessions with stakeholders from academia, healthcare and industry, and included lawyers, data protection officers, data managers, AI researchers, and diagnosticians. One milestone was the AIDA GDPR policy^14^, which eventually grew into the full data sharing policy. Following further review and discussion of the policy draft by the AIDA community and the Steering Group, it was appraised by a lawyer responsible for data protection at Linköping University, the AIDA host organization. In late 2019, the policy was published and made publicly available^4^.

The policy is not yet backed by formal authority from governmental agencies. Instead, its weight and credibility stems from the fact that it was created by a national community of professionals, being well equipped to interpret the legal and ethical requirements for data sharing in this domain.

## AIDA Data Hub Overview

The AIDA Data Hub was established to support the data sharing needs of the AIDA partners in academia, industry and healthcare. Data collection for the Hub is prioritized for areas with the highest potential for clinical added value, as identified by a Clinical Council. At a conceptual level, the AIDA Data Hub offers a service similar to the European Genome-phenome Archive^15^: facilitating FAIR data access for bona fide researchers. In this model, access to data is contingent on establishing the ethical and legal status of access requests. Verifying access can be time consuming, which makes data less accessible for research than it could be. Therefore, the hub is designed to include not only data storage but also pre-agreed and well-understood practices, where the core AIDA organization takes on a facilitating role (mediate contacts to data owners, assist with data transfer, etc).

Any researcher is welcome to request access to data in the AIDA hub. The licensing terms under which data may be shared and reused are provided along with the dataset descriptions, as well as contact information for further inquiries or access requests. For formal partners of AIDA, sharing is simplified: the recommendation is to use the pre-defined AIDA-BY license^16^, which is an adaptation of the succinct and permissive ISC open source license^17^ to address use for any purpose within AIDA (where scope and purpose of use are well-understood) provided that the dataset is cited in resulting publications. Any Swedish organization in the medical imaging research and development domain can become an AIDA partner.

**Fig. 1:**
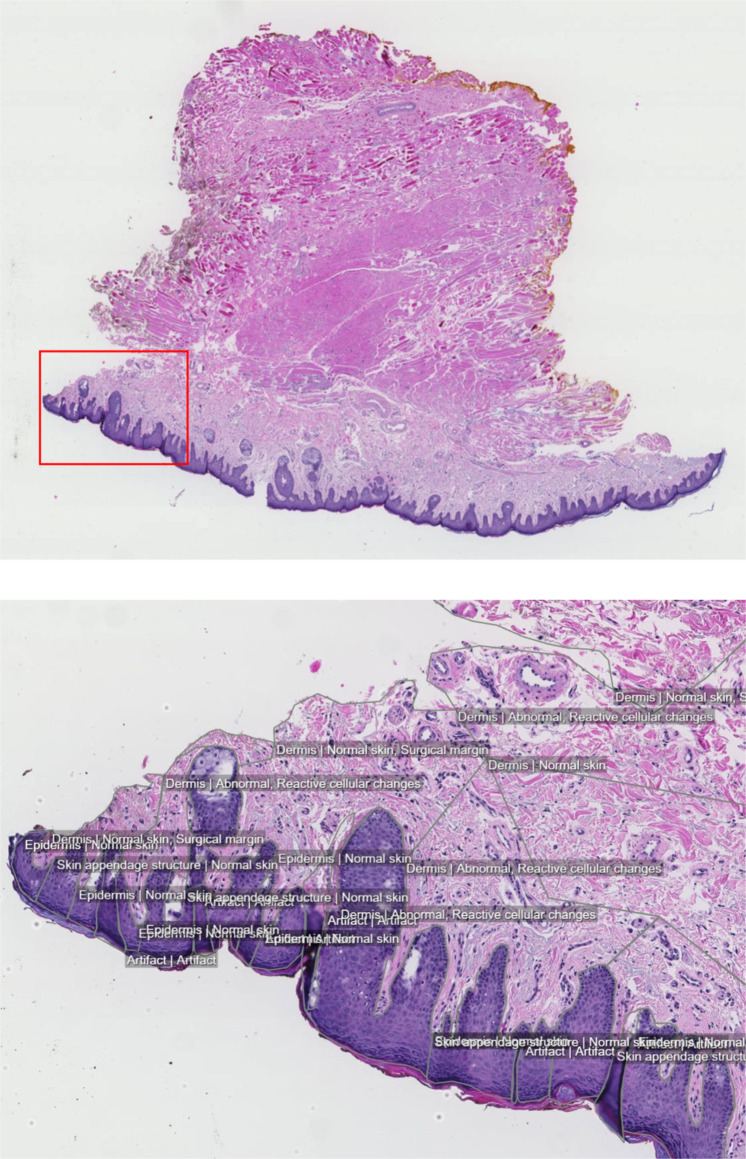
Whole-slide image from the DRSK skin pathology dataset shared on the AIDA Data Hub. The tissue section shown was stained with hematoxylin and eosin (H&E) and scanned as part of clinical routine. As described in an ethically approved research project plan, the data were anonymized and extracted to the AIDA Data Hub, where annotations were added by a pathologist. (**a**) Overview of entire tissue section. (**b**) Detail view corresponding to the red rectangle, showing the annotation granularity, similar for the entire section and all images in the data collection.

The hub includes researcher incentives, primarily by issuing Digital Object Identifiers (DOI) for shared datasets to make them citable. For increased visibility, shared datasets are advertised on search engine optimized DOI landing pages, which also helps to convey their reuse value and to highlight new collaboration opportunities. As an example, some images from the DOI landing page for the DRSK skin pathology dataset^18^ are included in Fig. 1, illustrating the quality and volume of the included expert-provided annotations. The DOI landing page also provides an overview description of the dataset and annotation strategy, including technical and clinical metadata. For increased findability, the AIDA Data Hub has a DOI and a landing page of its own at re3data.org^19^, which is a resource that systematically lists valuable data sources for research.

## Initial Policy Use

Although the policy was published only recently, there are already indications of a growing impact. The AIDA community, which includes most Swedish organizations working with AI in diagnostic imaging, has put the policy to use in many settings: facilitating data access for research, developing care provider practices of data extraction, writing of ethical review applications, as input to healthcare strategies for AI and data sharing, and as orientation for management researchers studying organizational AI practices.

The following are two examples of how the practices defined in the policy were employed in data sharing efforts. A non-AIDA researcher from a non-Swedish organization found the Data Hub website, and concluded that one specific dataset would be valuable for his research. He contacted the data owner, whose e-mail address is listed in the hub pages. The data owner checked that the request came from a legitimate source and that the intended use was within the ethical approval. A data sharing agreement based on the templates in the AIDA policy was signed by owner and recipient. The data owner asked the AIDA organization to extract the data from the hub to a temporary location for transfer, and once available he provided access to the requesting researcher.

A second example was when researchers from two AIDA partner organizations needed one of the datasets in the hub. Based on the pre-approval for within-AIDA use and direct access to the AIDA platform, they could download the data. The data were used in applied AI experiments and became part of a conference paper^20^, where the dataset was cited for the benefit of the dataset contributors.

## Concluding Remarks

Data sharing is fundamental for effective AI research in diagnostic imaging. The lack of clear answers on how to interpret generic legal and ethical requirements is an obstacle that we have addressed for the Swedish context with the AIDA data sharing policy.

Like the GDPR, this policy is written to be technology-agnostic, and as such there are many technology approaches that may satisfy the guidelines that it draws up. This allows implementations to let their respective use cases dictate the most appropriate technology choice -be it centralized or distributed or federated- based on the particularities of the data, as well as the specifics of the processing and the available resources for operating it.

It is, however, crucial to note that the AIDA policy is an interpretation and not an absolute truth. While we believe that we have achieved robust judgments as this is a best effort by a national community, we also acknowledge the need for continuous evolution of this best practice. Moreover, the conclusions currently reflected are not the only possible ones, and they should be challenged both by domain specialists and legal professionals. Technical advances may also necessitate re-evaluation, for instance regarding what means are *reasonably likely* to allow for re-identification. We hope that this initial AIDA policy is both an effective aid for researchers in the diagnostic imaging domain today, and will evolve and refine, and that it will provide a useful footing for similar efforts in policy development in other countries and disciplines.
